# Factors that influence enrollment in syringe services programs in rural areas: a qualitative study among program clients in Appalachian Kentucky

**DOI:** 10.1186/s12954-021-00518-z

**Published:** 2021-06-30

**Authors:** Umedjon Ibragimov, Katherine E. Cooper, Evan Batty, April M. Ballard, Monica Fadanelli, Skylar B. Gross, Emma M. Klein, Scott Lockard, April M. Young, Hannah L. F. Cooper

**Affiliations:** 1 Emory University Rollins School of Public Health, Atlanta, GA USA; 2 University of Kentucky College of Public Health, Lexington, KY USA; 3 Kentucky River District Health Department, Hazard, KY USA

**Keywords:** Syringe services programs, People who inject drugs, Rural Appalachia, Stigma

## Abstract

**Background:**

Enrolling sufficient number of people who inject drugs (PWID) into syringe services programs (SSP) is important to curtail outbreaks of drug-related harms. Still, little is known about barriers and facilitators to SSP enrollment in rural areas with no history of such programs. This study’s purpose was to develop a grounded theory of the role of the risk environment and individual characteristics of PWID in shaping SSP enrollment in rural Kentucky.

**Methods:**

We conducted one-on-one semi-structured interviews with 41 clients of 5 SSPs that were established in rural counties in Appalachian Kentucky in 2017–2018. Interviews covered PWID needs, the process of becoming aware of SSPs, and barriers and facilitators to SSP enrollment. Applying constructivist grounded theory methods and guided by the Intersectional Risk Environment Framework (IREF), we applied open, axial and selective coding to develop the grounded theory.

**Results:**

Stigma, a feature of IREF’s meso-level social domain, is the main factor hampering SSP enrollment. PWID hesitated to visit SSPs because of internalized stigma and because of anticipated stigma from police, friends, family and healthcare providers. Fear of stigma was often mitigated or amplified by a constellation of meso-level environmental factors related to healthcare (e.g., SSPs) and social (PWID networks) domains and by PWID’s individual characteristics. SSPs mitigated stigma as a barrier to enrollment by providing low threshold services in a friendly atmosphere, and by offering their clients program IDs to protect them from paraphernalia charges. SSP clients spread positive information about the program within PWID networks and helped their hesitant peers to enroll by accompanying them to SSPs. Individual characteristics, including child custody, employment or high social status, made certain PWID more susceptible to drug-related stigma and hence more likely to delay SSP enrollment.

**Conclusions:**

Features of the social and healthcare environments operating at the meso-level, as well as PWID’s individual characteristics, appear to enhance or mitigate the effect of stigma as a barrier to SSP enrollment. SSPs opening in locations with high stigma against PWID need to ensure low threshold and friendly services, protect their clients from police and mobilize PWID networks to promote enrollment.

**Supplementary Information:**

The online version contains supplementary material available at 10.1186/s12954-021-00518-z.

## Introduction

Syringe services programs (SSPs) are a proven [1] and highly cost-effective [2–5] intervention to reduce the risk of HIV and hepatitis C virus (HCV) transmission among people who inject drugs (PWID). Modeling suggests that expanding SSPs as HIV prevention interventions in the United States (USA) would cost about $25,000 per quality-adjusted life-year [QALY] gained, significantly below $50,000 per QALY benchmark of public health cost-effectiveness [6]. As for HCV prevention, SSPs may save about $364,000 per case averted [4]. Cost-savings of expanding SSP in combination with other evidence-based interventions may be particularly high for rural areas that have high prevalence’s of HCV and other drug-related harms [5].

Historically, SSPs have been scarce in the rural US though. In 2013, only 30 SSPs operated in rural areas, and their capacity and funding were lower than SSPs located in urban areas [7]. However, in response to the escalating epidemics of substance use disorder (SUD) and injection drug use (IDU)-related harms, including HCV, the state of Kentucky established more than 70 SSPs in five years following the legalization of this intervention in 2015 [8]. Almost half (*n* = 32) of these programs were opened in rural counties that were identified as experiencing or at high risk for IDU-related HIV and HCV outbreaks [9]. This unprecedented expansion of SSPs made Kentucky a US leader in the number of SSPs serving PWID in rural areas.

Despite rapid expansion, SSPs in rural areas may face challenges with PWID enrollment, which may hamper programs’ ability to address the epidemics of bloodborne infections at the population level. Lancaster et al. found that only 49% of PWID sampled in five Appalachian Kentucky counties had ever utilized SSPs, although 80% of the study participants had been recruited in three counties where SSPs were operating [10]. Allen et al. reported that almost 30% of PWID in a rural West Virginia County received sterile syringes from sources other than SSP [11]. To prevent or curtail outbreaks, a high proportion of PWID need to enroll and start using SSPs and other evidence-based programs. Modeling suggests that substantive and cost-effective reductions in HIV among PWID may be achieved if at least half of PWID population regularly receive SSP services [2]. Further, while SSPs may reach PWID indirectly via satellite and secondary exchange, in-person visits of PWID to SSPs are essential to link them to a comprehensive range of services, including HIV and HCV testing and treatment, as well as SUD treatment, wound care, housing and other social services [12–16]. However, literature on factors that may influence PWID enrollment into SSP services in high-stigma areas with no history of harm reduction programs is limited.

In this regard, an important question in studying SSP expansion in areas with no history of such programs is assessing factors that may accelerate or slow down enrollment into this program. Stigma is one such formidable barrier for PWID. It may take various forms, including public stigma (defined as negative stereotypes and judgmental attitudes toward PWID), enacted stigma (defined as overt ostracism and discrimination against PWID); anticipated stigma (defined as avoiding situations such as a visit to SSP that may disclose one’s drug use and expose one to enacted stigma); internalized stigma (defined as feelings of shame and unworthiness that affect one’s behaviors); and structural stigma (e.g., repressive drug policies and policing practices) [17–19]. Each of these forms of stigma either directly prevents PWID from engaging with SSPs and other health-related services, or fuels other forms of stigma and discrimination [10, 18–27]. Stigma may differentially impact sub-populations of PWID—for example, women who inject drugs may be subject to stronger stigma than men [28]. Stigma is an important barrier to services in rural Appalachia: PWID living in this region report that anticipated stigma is a reason to avoid attending SSPs or obtaining and carrying clean syringes [10, 20, 26]. Public stigma against PWID and related local opposition to harm reduction hampered access to drug-related services in the region, including preventing new SSPs from opening or closure of existing ones [21, 24, 25, 29–33].

Given the importance of enrolling a high proportion of PWID into SSP in high-risk settings and limited knowledge on barriers and facilitators of SSP enrollment in rural areas, we conducted a qualitative study among program clients in five rural counties in Appalachian Kentucky that had opened an SSP in 2017–2018. The study’s purpose was to develop a grounded theory of the role of the risk environment and individual characteristics in SSP enrollment among PWID. Since IDU-related stigma is prevalent in rural Kentucky, [10, 24, 29] we were especially interested in studying factors that may amplify or mitigate the role of stigma as a barrier to enrollment. The study findings may inform planning and implementation of SSP in rural areas.

### Theoretical framework

We explore this topic using grounded theory methods—a method of systematic exploration of raw data to infer major constructs and relationships among them to develop a theory explaining the phenomena of interest. While grounded theory originally prescribed the use of abductive logic without relying on a priori theories, constructivist grounded theory accompanies abductive logic with sensitizing concepts. These sensitizing concepts may include theories and frameworks from the literature to inform the development of the grounded theory [34]. Following the advice by Charmaz on sensitizing concepts, we used frameworks as “…points of departure from which to study the data” [34 p. 259], not as defining premises of our grounded theory. This helped us to align our analysis and grounded theory with the existing literature on drug-related risks and services. Specifically, Collins and colleagues’ Intersectional Risk Environment Framework (IREF), [35] an extension of Rhodes’ Risk Environment Framework (REF) informed our analyses. REF posits that drug-related risk behaviors, including service utilization, are the function of influence and interaction of external forces mapped by REF into environment domains (physical, social, policy, economical, law enforcement and healthcare) and levels (macro-, micro-, and, in some literature, meso-level). REF posits that stigma is a feature of macro-level social environment, rooted in negative stereotypes, norms and beliefs prevailing in rural communities, and intertwined with policy and law enforcement (“War on Drugs”) environments [29]. IREF extends REF by conceptualizing an intersecting social location of an individual (a set of individual characteristics, such as age, gender, race, defined by the systems of oppression or privilege) as a mechanism mediating and modifying the impact of risk environment on individual health outcomes [35].

## Methods

### Study design

We employed a qualitative multiple-case study design; individual SSPs in each of the five study counties were treated as separate cases [36]. To preserve anonymity of the study participants, we do not mention the county names. A case study design is suitable for studying real-world phenomenon (here, SSPs) in situations where the boundaries between the phenomenon and the context (PWID networks, service providers, communities) are not clearly delineated. In multiple-case studies, each case (SSP) is considered a replication of an “experiment” generating data to develop and iteratively refine a theory explaining the phenomenon [36–38]. Prior to conducting interviews with SSP clients, we reviewed the interview guides with SSP managers, staff and directors of health districts overseeing local health departments in the study counties.

### Setting

The study gathered data from SSPs in five counties in Appalachian Kentucky. These SSPs were managed by county health departments and located within county health centers; no mobile SSPs had been launched in these counties at the time of data collection. Service provision was not uniform across the SSPs: some were open during most business hours four or five days a week, while others were open only a couple of hours one or two days a week. Some SSPs had more strict syringe exchange policies than others (e.g., at some SSPs clients could exchange up to 200 syringes per visit, while in others a cap was set at 20 or 40). All counties were rural, ranking between 7 and 9 on USDA Rural–Urban Continuum Code (1—most urban, 9—most rural) and with population density ranging between 23 and 85 people per sq. mile [39, 40]. As elsewhere in rural Appalachia, these counties struggle with high levels of health disparities, poverty and unemployment as the result of decline in coal mining and agriculture [40–43]. As in many US rural areas, these counties were geographically remote and residents were often isolated; public transportation was limited and healthcare facilities operated under resource constraints [41, 42, 44, 45]. The opioid epidemic that hit the area hard has been accompanied by a high prevalence of HCV, IDU, overdoses and condomless sex [46–50]. Four of the five study counties were determined by CDC to be experiencing or at high risk for HIV and HCV outbreaks due to IDU, with three of the counties ranking among the top 30 most vulnerable to such outbreaks [9]. Previous studies reported dense and overlapping social and drug use networks, often including members of the same families, as a key characteristic of the risk environment [51–53]. These dense social networks and intergenerational ties are the main feature of communal resilience, defined as an ability of individuals, families and communities to harness their social support systems to overcome or cope with multiple challenges of everyday life in these impoverished and underserved rural areas [51–53].

#### Sampling and recruitment

SSP clients were the population of interest for this study. Eligibility criteria included being aged 18 or older; receiving syringes at one of the five SSPs at least three times in the past three months; and residing in the county where SSP operated for at least six months. SSP clients were recruited in three ways: (1) via SSP staff, who referred interested clients to research assistants (RAs); (2) RAs approached SSP participants at SSP premises; and (3) RAs contacted participants of the parent study cohort who reported using SSP services at the time of the survey and had agreed to be contacted about future research. Our final sample comprised of 41 PWID—program clients. All participants provided verbal informed consent.

#### Data collection

We collected data via in-person one-on-one semi-structured interviews. The interview guide covered the following topics: injection practices prior to attending the SSP and unmet needs; the processes of becoming aware of SSPs in the county and making the decision to start participating in it; and barriers and facilitators to the first SSP visit. Interviews were audiotaped with participant consent. In addition, we conducted a mini-survey to collect data on participants’ sociodemographic characteristics, drug use and SSP service utilization patterns. Participants were offered $20 cash as a compensation for their time. Interviews were conducted between December 2018 and January 2020 (all SSPs had been operating for more than one year by the start of the interviews). The study has been approved by Emory University Institutional Review Board (protocol IRB00107426).

#### Data analysis

A constructivist grounded theory approach was used throughout the data collection and analysis phases [34]. Interview guides were iteratively reviewed and revised based on the study findings and emerging grounded theory. Interview records were transcribed verbatim and coded in NVivo 12.0 software (QSR International).

We started with a *within-case analysis*, in which we analyzed data from each county (there was one SSP in each county), one at a time. The within-case analysis had three stages—open, axial and selective coding—and each stage employed constant contrast and comparison techniques. We documented our analyses in memos for each county. After reading each transcript multiple times to immerse ourselves in the data, two coders started the *open coding* process independently by labeling concepts in four initial transcripts and developing the codebook. Concepts with similar meaning or pertaining to the same phenomena were combined into higher order categories. We revised the codebook iteratively as new codes and categories emerged while coding the remaining transcripts. Subsequently, every fourth transcript was double-coded to enhance reliability; discrepancies were resolved by having a third person code the discrepant text and subsequent discussion in the team. At the *axial coding* stage, we linked categories to each other by developing relational statements. During *selective coding,* we selected the central category (here, “stigma as a barrier to SSP”) most saliently related to our research question. We consulted our theoretical framework (the Intersectional Risk Environment Framework) when we labeled the major categories of the theory. For example, we grouped into IREF domains (e.g., policy, social, healthcare and law enforcement) the external factors related to the central category. This three-stage process was repeated for each county.

In the *cross-case analysis* stage, we refined the emerging grounded theory by comparing and contrasting major categories and the relationships between them across the counties (cases). Specifically, we looked for literal replications (similar findings) and theoretical replications (contrasting findings predicted by the theory) of the core categories and the relationships between them. This contrast and comparison process was recorded in the final memo, which encompassed findings from all counties and described the grounded theory reflecting differences and similarities across the counties.

## Results

### The study participants—SSP clients

Mini-surveys were completed by 37 out of 41 participants (four participants did not complete the mini-survey; Table 1). Our full sample consisted of 18 men and 23 women (according to the screening data). The subsample that responded to the mini-survey included 17 women (45.9% [unless indicated otherwise, the denominator for percentages in this paragraph is 37—the number of the mini-survey participants]) and 20 men (54.1%), both young and middle-age adults, with mean age of 38 years. Almost all participants self-identified as non-Hispanic white, consistent with the local racial/ethnic composition. The most commonly used drugs were methamphetamines (67.6%), marijuana (51.3%), prescription opioids (40.5%) and prescription sedatives (40.5%). Methamphetamines were also the most commonly injected drug in the three months prior to the interview (64.9%), followed by prescription opioids (43.2%) and heroin (32.4%). On average, participants started visiting study SSPs more than a year before the interview time (mean = 13.2 months). According to the individual interview data, about half of our participants reported postponing their first visit to SSP for a month or longer after first hearing about the program, and several of them (7 out of 41) waited for six months or longer.

**Table 1:** Demographic characteristics, drug use practices and length of SSP participation (*N* = 37)

Characteristic	*n* (%)*
Age (mean, SD)	37.6 (8.9)
*Gender*	
Men	17 (45.9)
Women	20 (54.1)
*Race/Ethnicity*	
White non-Hispanic	35 (94.6)
Other	1 (2.7)
Refused to answer	1 (2.7)
*Commonly reported drugs used (past 3 months)*	
Methamphetamine	25 (67.6)
Marijuana	19 (51.3)
Prescription opioid painkillers	15 (40.5)
Prescription sedatives	15 (40.5)
Heroin	12 (32.4)
Gabapentin	12 (32.4)
Suboxone	11 (29.7)
Fentanyl or Carfentanyl	6 (16.2)
Cocaine	5 (13.5)
*Commonly reported drugs injected (past 3 months)*	
Methamphetamine	24 (64.9)
Prescription opioid painkillers	16 (43.2)
Heroin	12 (32.4)
Suboxone	11 (29.7)
Fentanyl or similar	5 (13.5)
Cocaine	3 (8.1)
Number of months since first visit to the SSP (mean, SD)	13.2 (7.8)

^*^% are calculated for participants who participated in the mini-survey (*n* = 37)

### Overview of the grounded theory

**Fig. 1:**
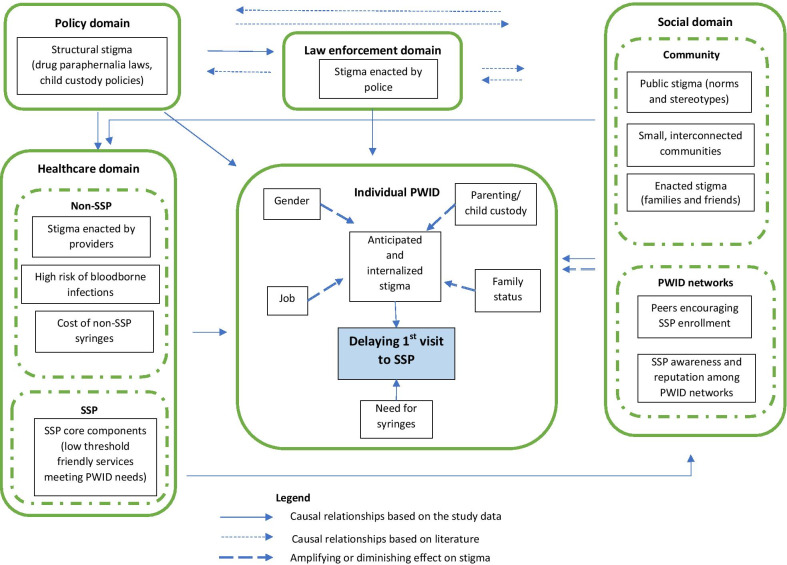
Model of syringe service program initiation by people who inject drugs (following the Intersectional Risk Environment Framework)

According to our grounded theory (Fig. 1), prior to visiting SSPs PWID were in high need of free sterile syringes due to the high cost and limited access of syringes outside of SSPs. Still, PWID hesitated to visit SSPs because of stigma. They feared persecution by law enforcement, and judgment and ostracism by friends, family, healthcare providers and by the general public. We categorized this fear as *anticipated stigma*. PWID also hesitated to enroll in the SSPs because of shame and feelings of unworthiness related to their drug use; we categorized these experiences as *internalized stigma*. According to our emergent grounded theory, anticipated and internalized stigmas were mitigated or amplified by individual- (gender, child custody, social status), network- (family, peer support) and organizational-level (low threshold-SSP services) factors.

We start illustrating the theory by describing two cases—one participant who waited for a long time after hearing about the SSP, and another who started visiting the SSP soon after learning about the program. These cases provide real-life illustrations of how network, organizational and individual-level factors influenced our participants’ decision to start visiting SSP (Box 1). Case descriptions are followed by detailed and structured descriptions of the findings elucidating the main categories of the grounded theory and the relationships between them.

Box 1. Deciding to visit SSP—short and long lag (delay in the first visit to SSP) **Case # 1 (short lag):** Todd (pseudonym) is 37 years old, has young children and lives in county B. Prior to enrolling in the SSP, Todd used to buy syringes from pharmacies and on the black market. He reused his own syringes after cleaning them, and did not practice receptive syringe sharing. He learned about the program via his social network—a friend told him about it. Unfamiliar with SSPs, he initially hesitated to visit the program for about two weeks because of anticipation of structural stigma—he feared that police may arrest him for drug possession or under some other pretext. He was also worried about losing custody of his children if SSP staff reported him to child protection service:

I'm like oh man, … they’re going to arrest you when you go up there [to SSP] … They’re going to get you for a PI or public intoxication, I mean DUI or try to hit you with possession or something. Because I had a nephew get a county year in jail for having a syringe… And, so I thought, no man, you know you all crazy to do it. […] I thought maybe – I have kids, right, so I thought well, I’ll come up [to SSP], I go up and sign my name…, [then SSP staff] want [to notify] social workers, you know what I mean, with kids and whatnot.

However, his social network members who had visited the SSP helped him overcome his concerns when he talked to them and found out no one had been arrested:

Interviewer (I): So, after you first heard about it, about how long did it take you to decide and go to the program?

Participant (P): … about two weeks, first week I was a little bit hesitant about it, … talked to somebody that’d been here and went [to SSP]. And seen that they wasn’t in jail, and I thought alright, I’ll go.

**Case #2 (long lag):** Nancy (*pseudonym)* is 41 years old, a mother of two children (one of them young), has two brothers who also inject drugs, and lives in county A. Nancy hesitated for six months before deciding to visit the program. Prior to SSP enrollment, she too used to buy syringes on the black market, cleaning them with alcohol and bleach; she also reused her own syringes. To the best of her knowledge, Nancy did not inject with borrowed syringes before attending the local SSP because she was concerned about her health. Nancy had no prior awareness of SSPs and heard about the program in her county via her social network: a close friend who was an SSP client told her about the program. Internalized and anticipated stigma were the main barriers delaying SSP enrollment. A long-time resident in her small rural county, Nancy was ashamed and embarrassed at the thought of potentially bumping into an acquaintance there:

… this is a very small town. I’ve lived here my entire life. Just the fact of running into [people I know] – I just find it really embarrassing. I find it mortifying, really.

Nancy was also concerned about police seeing her visiting the program. She discussed the possibility of visiting the SSP with her friend and her boyfriend, who encouraged her to enroll to the program. Eventually her need for syringes, both for herself and her brothers, overcame her concerns about stigma, and motivated her to enroll. One day, six months after first hearing about the program, Nancy mustered the courage to visit the program unaccompanied. After enrolling in the SSP, Nancy encouraged her boyfriend to start attending too.

I: Okay. So then what kind of led you to the decision to come? You said it took you about six months, and you were talking to your friend.

P: The fact of having clean syringes – that was a very important thing. It was a lot safer that way.

I: So, who all did you talk about coming here with?

P: Well, she’s a really close friend of mine; and my boyfriend at the time – my boyfriend – we talked about it. I came first. I just decided one day. I couldn’t make myself come, actually; and then one day I decided to try it, and I came without letting anybody know. I just came hoping for the best, that there wouldn’t be a lot of people. I didn’t know what to expect. I came, and then I told [my boyfriend] about it; and then he started coming, too.

### Healthcare environment—scarcity of syringes outside of SSP

Unmet need for free and sterile syringes^1^ was the main reason that PWID started visiting SSPs. Lack of access to syringes and their high cost outside of SSPs were main features of healthcare environment that drove high need for sterile syringes among PWID. In the absence of sterile syringes, PWID engaged in risky injecting practices.

Prior to visiting the SSP, PWID in all counties had highly unreliable access to syringes because it was difficult to purchase them over the counter. Almost all participants prior to enrolling in the SSP bought syringes on the black market from people who sell drugs or from people with diabetes. Black market syringes cost about $5 per syringe,^q1^^2^ a price too high as mentioned by many participants. In addition, syringes bought on the black market might not be sterile:

P: You’d never know when you’d be able to get a new [syringe] or not. Some people you’d have to watch out, because they would try to sell you some; and they’d be used. […] the new ones – they’ve got an air pocket that you have to pop when they’re brand new. […] It’s not on the used one.

*- a man from county A*

Several participants mentioned buying syringes without a prescription in pharmacies located in large cities outside of the study area. The cost of syringes in these pharmacies was much lower—$13 per 100—compared to the black market^q2^. However, PWID had to spend considerable time driving to large cities to buy syringes from pharmacies, and urban pharmacies were not always a reliable source of syringes since pharmacists might have refused selling syringes at their discretion:

But like back in the day when you used to be able to buy them at Walmart…in Lexington. We had to drive … to Lexington to get a dollar and a half pack of rigs. […] But then we started coming and getting too many and they shut us down.

*- a woman from county E*

Limited access to syringes led to risky injection practices—almost all participants reported reusing their own syringes and some participants reported engaging in receptive syringe sharing (i.e., using a syringe after someone else) prior to SSP enrollment. Reusing old syringes made the needles dull and burred, resulting in injured and scarred veins and soft tissue, also causing pain and discomfort. A man from county B described using a dull needle as “…*sticking a railroad spike in my arm*.”

### Stigma and hesitation to visit SSP

As shown above, many participants in our sample had unmet demand for sterile inexpensive syringes. Nevertheless, about half of our participants reported postponing their first visit to SSP after learning about the program for a month or longer. All participants who delayed their visit to the SSP and some participants who started visiting the SSP relatively soon after hearing about it expressed reluctance to visit the program because they anticipated various forms of drug-related stigma. Here, we describe the most common forms of stigma that caused concern and delayed SSP visits: a) fear of persecution by law enforcement and other legal repercussions; b) anticipation of stigma enacted by public, family and friends if their drug use were disclosed; and c) fear of possible mistreatment by SSP providers. Importantly, participants’ accounts also elucidated how individual and environmental factors mitigated or amplified the fear of stigma as a barrier to visiting SSP. We describe the various types of anticipated stigma and factors modifying its effect in the subsections below.

#### Fear of law enforcement

PWID feared that visiting an SSP would provide evidence of illicit drug use, and so were reluctant to start visiting SSPs. Many participants were afraid that the SSP was a police setup to identify and target PWID. Some participants initially believed that SSP staff collaborated with police and shared information about clients. One participant refused to visit the SSP when she heard about it, since she believed police would arrest PWID visiting the program.

They was a friend of mine that told me that this program was going on; and I said, my God. I said every one of us in this town is going to go to jail. […] I thought it was just a joke really; and [my friend is] like, no, they give [syringes], and then you take them back every time you – I said you’ve lost your mind, because when you go down there and do there, there’s the law going to be sitting there, take your ass to jail. I said well, when you go to jail, don’t say I didn’t tell you so.

*- a woman from county C*

This fear of police was based on past persecution of PWID by law enforcement, as reported by many participants. While most participants’ accounts of stigma enacted by law enforcement focused on police officers stopping, searching, arresting and confiscating syringes from SSP clients, some participants also recalled episodes of arrests and imprisonment for paraphernalia charges that occurred prior to the SSP’s opening. Persecution of PWID by police was not a mere act of enforcing the law, but also manifestation of stigma against PWID since many police officials shared negative attitudes toward PWID. A participant recalled the local sheriff’s political platform based on “cleaning up” the county by incarcerating people who use drugs:

P: Well, during his campaign he kept hollering that he was going to “clean up” [our] County, he was going to put all the drug addicts in jail, he was going to put all the drug dealers in jail, and he just looks down on us. That we're nothing, more or less...

- a woman from county C

#### Fear of public stigma

Anticipation of public stigma was a common reason for delaying SSP enrollment. PWID feared that their family members, friends, employers and other members of the general public would find out about their drug use if they visited SSP. According to participants, public stigma toward PWID was prevalent in the study counties. PWID were perceived as people of lower value who “do not matter,” who engaged in crime and damaged the local communities.^q3^ In this setting, PWID wanted to hide their drug use, even from their family members and friends. They worried that visiting the SSP might inadvertently reveal their drug use and subject them to ostracism and ridicule by kin and kith. Some participants mentioned visiting SSPs in another county to avoid being seen by familiar people. In some cases, PWID concealed their drug use from family members to spare them from embarrassment and stigma by association. A participant mentioned that while her adult children might know she used drugs, she did not want them to be confronted about it by others:

[…] like I said, my children were of age, I didn’t want them to find out [about my drug use]. In the same talking, my daughter’s a social worker she probably knows more than I think she knows. She’ll be a [healthcare professional] coming this spring when she graduates this year… So, she probably knows a whole lot more than what I think. But I just didn’t want them exposed to that. And I didn’t want them to have people coming up and say, hey, we’ve seen your mom with at the needle exchange. So that’s the only [concern I had about visiting SSP].

*- a woman from county B*

Living in small tight-knit communities where many people know one another and rumors spread fast amplified fear of public stigma. In some cases, PWID were reluctant to visit the SSP since their friends or acquaintances worked at county health centers where SSPs were located. PWID feared that these people might tell their families and friends about their SSP use:

Some people don't want nobody seeing you come in to get [syringes], some people’s parents or kinfolks work [in the Health Center], or lives next to a person that lives here and they don’t want them to know.

*- a man from county C*

Anticipation of public stigma was seemingly compounded by internalized stigma, as some PWID avoided SSPs because it reminded them of their perceived failure and moral weakness.^q4, q5^ A participant was embarrassed to visit SSP because she was ashamed of her drug use:

I: Were you worried about anything about trying this program out for the first time?

P: Just ashamed and didn't want no one to know me, which no none here knows me much anyway, but that's not the point. I just - there's always going to be shame. I'm 48 years old. I'm a grandma. I didn't get it over this to kick it in the butt - kick it to the curb, you know. And I’m trying my best to do so. The day I don't have to come here and get a pack of needles I'll be so happy.

- a woman from county D

PWID might also delay visiting SSPs due to anticipation of stigma by SSP staff. As indicated above, living in a small community increased chances of PWID seeing someone familiar working at a SSP or health center. Some participants believed that SSP staff would share clients’ information with law enforcement or treat clients with prejudice.^q6^ Fear of stigma by SSP staff might be based on negative experiences of PWID who were stigmatized by other healthcare providers. A participant recalled a disparaging remark from a local clinic staff:

[Healthcare staff] treat you like you're nothing. Like you're trash. … You look a certain way, whether you're a user or not, you're being treated as one. This area is real bad for it. I know when I had MRSA, I had a problem with one of the staff [who] made a comment to me that, "You're nothing more than an old dopehead drug user, and I don't even know why we're fooling with you."

*- a woman from county D*

Distrusting healthcare systems, some participants simply did not find the idea of an SSP credible initially. One of the participants who delayed joining SSP did so due to his disbelief in the idea of SSP:

I: What did you think about the program when you first heard about it? Or how did you feel about the program?

P: I was sketchy about it at first, until I started actually going…. I just thought it was too good to be true or something. I don't know. If something sounds good, it's usually not a good thing, you know?

*- a man from county A*

### Factors modifying stigma as a barrier to first visit

While analyzing stigma as a reason for the first visit to SSP, we identified several categories that amplified or mitigated the level of anticipated stigma or helped the participants to overcome strong anticipated stigma. These factors, including individual characteristics as well as factors belonging to social (PWID networks) and healthcare (SSPs) domains, are discussed below.

#### Individual-level factors modifying anticipated stigma

The data suggest that **gender** is an important individual-level factor modifying the effect of stigma on delaying the first visit to the program. Notably, participants’ gender may both amplify and mitigate stigma as a barrier. SSPs were located in county health centers, and several participants mentioned that they knew it as a place where women, including women with children, were regular visitors. Some women appeared to be more comfortable visiting these SSPs than men, as they found it easier to conceal their visit to an SSP as a visit for other purposes^q7^:

I: What concerns did you have about coming to the program?

P: I didn’t have any. I just don't want people to know what I do. I make up a story about something else that I do [in the county health center], but I just don't want anybody to know.

I: Do you mean you tell them you're at the health department for something else?

P: Yeah.

*- a woman from county C*

The role of gender was complex, however, and data also suggest that women, in particular mothers, might be more vulnerable to stigma, because society expects them to care for children and drug use was seen as not compatible with motherhood:

I: Do you think there are any particular kinds of people like women or younger people who might be less likely to come to the program?

P: Yes, mothers, young mothers or mothers in general, who just don’t want people to look down upon them because they’re a mother or look down upon their kids somehow you know what I’m saying. They just don’t want people to think bad of them, like, I don’t have [to be seen as] a bad mom because I use.

*- a man from county E*

PWID with young children were also susceptible to structural stigma, since disclosure of drug use status might jeopardize their **child custody**.^q8, q9^ Notably, not only women, but also several men in our sample expressed concerns about losing child custody or exposing children to stigma by association:

P: My kid ain’t never seen nothing, she never see nothing. I don’t even [use drugs] when I’m at the house. I always do it when I’m gone.

I: Where you worried that your daughter would know that you came [to SSP]?

P: Well, just maybe, maybe grown people would get the information [about my drug use], you know what I’m saying. Maybe cause me some problems … Maybe [they will] call social [child protection] services or something … You got to worry about stuff like that, especially round this town.

– *a man from county B*

Disclosure of drug use status might result in loss of social status or damage to personal relationships of PWID**. Higher social status** (including having higher income, being employed, older age or high status in family hierarchy) thus is a factor increasing individual’s susceptibility to higher internalized stigma and fear of anticipated sigma. Participants mentioned that some categories of professionals whose careers depended on their reputation might be at higher risk of losing their job if someone saw them at SSP:

A lot of people with, you know, jobs – like certain kind of jobs don't come to the program because, you know, if the wrong person's seen them at the program, then they're more likely to get fired…

I: What kinds of jobs would you say?

P: Like [a registered nurse]. Say like any kind of public official definitely ain't going to come in. Just professions like that. I mean, they're – that's for sure not going to happen, you know.

– *a man from county B*

#### Social domain—network-level factors modifying anticipated stigma

While PWID might hesitate to visit SSPs lest their social network members learned about their drug use, as shown above, PWID networks also facilitated SSP enrollment by advertising SSP services and addressing misconceptions about the program. Members of PWID social and drug using networks played a significant role in reducing anticipated stigma among their peers and encouraging enrollment to SSP. Learning about the SSP from people they knew was a reassurance for PWID who were hesitant, especially if the person sharing the information about the SSP has previously attended the program.^q10, q11^ Many participants in our sample were accompanied during their first visit by their family members, partners or other trusted peers who already started visiting the program.^q12^ Some PWID hesitant to visit SSP asked their peers or non-PWID friends or family members to visit SSP to check if it is a safe and legitimate service. This happened particularly often when SSPs had just opened and their reputation among PWID had not been established yet:

I said you go on and sign up for me and get your little things. What you want to do, you go over there and get and I said, let’s see if you make it back down here or not. I thought it was just a joke really […] I sat down there [near SSP] and never moved from it.

- *a woman from county C*

Gradually, SSPs’ reputation of safe and legitimate services grew among PWID networks over time, as more PWID spread positive information about the programs. This was self-perpetuating: many participants also mentioned that after they started visiting SSP, they encouraged their friends to come to the program. Some of them gave out new syringes from SSP to reassure their hesitant peers:

I: So, since you’ve started coming here, have you encouraged other people to come?

P: Oh yeah, word of mouth is the best thing you’ve got.

I: What do you usually tell them?

P: Tell them that -- you know, I’ll give them a couple [of new syringes] and switch them if they’ve got some old ones and stuff. Look, just walk down the street. They aren’t going to bite you, they won’t hit you, you know, I’ve been in [SSP]. They aren’t going to arrest you. So why not? You haven’t got nothing to lose really.

- *a man from county E*

##### Healthcare domain—SSPs countering anticipated stigma

The provision of low threshold, anonymous and PWID-friendly services by SSPs was a key for their positive reputation among PWID, eventually helping to assuage their fears and concerns related to visiting SSP. All SSPs in our study took precautions to protect clients’ privacy and confidentiality, lest stigma prevents them from enrolling to the program.^q13^ For example, SSPs’ location in county health centers allowed their clients to blend in with other health center patients. Importantly, all SSPs issued program IDs to their clients to protect them from arrest or from police confiscation of their syringes. This was a key motivation for many PWID to start attending an SSP:

I: So then what kind of things would you say to these people to kind of talk them into maybe giving [SSP] a try?

P: I’m thinking I ended up convincing like maybe one or two. The card they give you, you know, showing that you’re part of the program. So, if you get pulled over by the cops and stuff, as long as you don’t have, you know, the paraphernalia associated with it, you know, you can, you know, skate by with it. You know, that card was beneficial so they knew that if they got caught with rigs in the car, you know, traveling back home or something, you know, they’re good. You know, they don’t have to worry about nothing. I think that was the key to, you know, getting a couple of my friends to start coming here.

*– a man from county A*

Personal awareness of or experience with SSPs also helped participants to avoid or overcome hesitation to visit the program. Participants who were aware of SSPs prior to their first visit, especially those who visited SSPs in other locations, knew about the program purpose and confidentiality protection measures. These participants anticipated much less stigma and tended to visit SSPs sooner.^q14^ We found no evidence that variations in SSP operations (e.g., hours) were related to variations in enrollment across programs.

### Negative cases

Contrary to the numerous accounts of anticipated and internalized stigma, there were several participants who did not internalize stigma related to drug use (negative cases). Across the study counties, several participants were not worried about others finding out about their drug use and visited a SSP soon after learning about it.

I: So, did you have any concerns about trying the program out? Anything that made you a little bit hesitant about coming?

P: No. At least get it over with, put this on that. I said I don’t give a damn who knows it. You can put it in the paper for all I care. That’s just the way I am.

- *a woman from county D*

## Discussion

To effectively address epidemics of drug-related harms, SSPs opening in areas such as rural Kentucky with limited access to sterile syringes and high prevalence of IDU and HCV need to reach a high proportion of PWID. Drawing on empirical data and IREF, we developed a grounded theory conceptualizing how the interplay of PWID social locations (individual-level characteristics) and various domains of the rural intersectional risk environment produces and influences stigma as a major barrier to SSP initiation. The role of stigma as a barrier to SSPs and other services targeting PWID in the USA, including rural Appalachia, is well documented [10, 17, 27, 54]. For example, 19% of PWID surveyed in Appalachian Kentucky cited fear of stigma as a barrier to SSP enrollment [10]. The most prominent manifestation of anticipated stigma in our study was fear of police persecution, a theme recurring in other publications on rural Appalachia [20, 55]. Enforcement of laws criminalizing possession of drugs and injecting equipment is a well-documented structural determinant of health. Past research in Appalachia and elsewhere has found that PWID who fear arrest tend to avoid SSPs and other HIV prevention services, or are reluctant to carry injecting equipment [20, 22–24]. Furthermore, fear of arrest, having been arrested, police confiscation of syringes, not carrying or buying syringes and rushing injections for fear of police are linked to higher rates of HIV [22, 23].

Our findings corroborate this literature, showing how various manifestations of stigma—anticipated, internalized, enacted and structural—make PWID reluctant to visit SSPs despite high unmet need for sterile injection equipment. The most important and novel contribution of our grounded theory to the stigma literature, though, is conceptualization of meso-level risk environment domains and individual’s *social locations* as factors that amplify or mitigate the role of stigma as a barrier to SSP services in rural settings.

Our grounded theory suggests that *social networks* are significant features of PWID’s meso-level social domain, influencing the impact of stigma as a barrier to SSP services. Dense family and broader social networks, a mainstay of rural Appalachians’ social support system [52, 53, 56], may play both negative and positive roles in promoting enrollment in SSPs. Fearing loss of this support system, PWID may decide against visiting the SSP, since it is hard to keep these visits private in small and tight-knit rural communities, and because SSP are run by county health center staff (the latter resulting from restricting SSP legislation in Kentucky, as discussed below). On the other hand, PWID’s peer networks may mitigate anticipated stigma by encouraging PWID to start visiting SSPs. In rural areas, PWID networks are also indispensable in informing their peers about the programs, since lack of awareness about SSP is one of the major barriers to enrollment [10]. The positive role of PWID peer networks in promoting SSP use is documented in the literature, including a study among PWID in rural Kentucky [26]. Globally, SSPs often capitalize on PWID networks by actively involving PWID in service provision, including outreach work and secondary exchange [57, 58].

Importantly, IDU social network members may encourage PWID to visit SSP if only these services are worth visiting. This analysis found that the positive reputation of SSPs among PWID networks was built and maintained by evidence-based implementation of services by staff at the local SSPs (meso-level *healthcare domain*). As attested by our participants, SSPs operating in these counties attracted PWID by meeting their needs in a low threshold, confidential and friendly manner. Specifically, the programs offered free syringes and other injecting equipment (both directly and via secondary exchange), provided services anonymously, protected clients’ privacy and treated them with dignity. SSPs also help to reduce the impact of structural stigma by issuing program IDs to protect their clients from police searches, syringe confiscation and arrest. These findings on the role of programs and social networks in reducing stigma and promoting access to services contribute to the growing acknowledgement of *meso-level* in REF literature, [59] showing how these factors absorb the impact of negative forces such as stigma acting at other levels of environment.

Notably, we found no evidence of a relationship between SSP characteristics and enrollment delays. Characteristics of SSP service provision mentioned in the literature [26, 60], (e.g., hours of operation, syringe exchange rules, lack of mobile SSPs or range of available services other than sterile syringes) did not seem to factor into our participants’ decision to enroll to the SSP. Perhaps these characteristics were not as important for the first visit to the program as they might be for continuous SSP attendance, or for other program outcomes such as client satisfaction or meeting their needs in services.

Similarly, our analysis did not identify factors pertaining to other domains of the rural risk environment that might have affected delayed enrollment. For example, geographic remoteness, lack of public transportation and costly private transportation for impoverished residents are well-known impediments to accessing drug-related and other health care services in rural Appalachia, but were not mentioned here [24, 55, 61–64]. Most likely, these prominent features of the rural risk environment may affect continuous attendance of SSP rather than PWID’s decision to visit the program for the first time.

Our interviews did not elicit much information on the impact of legal environment on SSP enrollment. The state of Kentucky does not fully exempt SSP participants from drug paraphernalia charges, [65] which may raise additional concerns among PWID contemplating SSP enrollment. Kentucky’s SSP legal framework limits syringe distribution to health departments and requires approval of the new programs by local authorities, effectively preventing SSP establishment or limiting its scope of operations in counties where municipal bodies disapprove of SSPs [66]. This legislation also prohibits community-based organizations (CBOs) from operating SSPs, which may create additional barriers for engaging PWID. PWID, however, did not raise concerns relating to these issues. We did identify though several *individual-level factors* or, following IREF terminology, *social locations*, that may interact with stigma as a barrier to SSP initiation. *Gende*r is one such characteristic. While our sample had roughly the same number of men and women as parents or custodians of underage children, women in the USA are more likely to have custody over children than men, [67, 68] and, in case of women who inject drugs, to have child custody-related concerns about visiting SSP. Literature also shows that women who inject drugs, in particular mothers, are subject to harsher judgment by the society as compared to men, and may be more likely to avoid visiting harm reduction services [69]. Importantly, the aforementioned survey among PWID in Appalachian Kentucky found that men were more favorable toward SSPs being located in county health centers than women were, while women were more likely to prefer local health department staff as SSP service providers [10]. These findings suggest that while women are warier to visit services in publicly visible locations, they prefer service providers associated with serving women and children.

*High social location*, including employment or position in a family hierarchy, may have shaped delays in first visits: people who were higher status may have been reluctant to attend the SSPs because of status loss, a component of stigma. As conceptualized by Link and Phelan, status loss, or “downward placement of a person in a status hierarchy” [70 p.371], becomes a source of discrimination. Individuals with higher position in the social hierarchy may also fear not meeting societal expectations affixed to their status [71]. Consequently, for people of higher status disclosure of their participation in stigmatized services is associated with failing societal expectations and facing discrimination.

*Strengths and limitations* of our study can be analyzed via the prism of Maxwell’s [72] validity framework. We enhanced descriptive validity (i.e., accurately capturing the content of participants’ accounts) by audio-recording the interviews and transcribing them verbatim. Interpretative validity (i.e., accurately conveying the meaning of participants’ accounts) was strengthened by joint development of codebook and reconciliation of coding discrepancies by the study team members. The steps to improve theoretical validity (i.e., the extent to which the grounded theory accurately reflects the phenomena of interest and the relationships between them) included having a relatively large and diverse sample of SSP clients; consulting theoretical frameworks widely used in research on PWID; refining grounded theory against findings across five counties (cases). However, the theoretical validity of our study may be undermined by not including PWID who never visited SSPs. Overall, our grounded theory has limited applicability to SSP in urban settings due to lower anti-PWID stigma and higher level of SSP clients’ anonymity (or lower public visibility) in urban areas.

### Public health and research implications

As suggested by our grounded theory and related literature, the impact of stigma on expanding SSP enrollment and coverage may be reduced at meso-level by following best practices of SSP implementation, including those implemented by SSPs in our study. Based on our findings and global literature on SSP, we suggest a range of recommendations for rural SSPs in Kentucky and beyond. As demonstrated by SSPs in our study, anticipated stigma by law enforcement may be addressed by program management and staff early by engaging local law enforcement to protect the program participants from paraphernalia charges and other forms of police harassment. Specific protective measures include issuing program IDs, reducing patrolling of the areas surrounding SSPs, and anonymous service provision. Law enforcement officers may be sensitized to the needs of PWID and importance of harm reduction approaches, as demonstrated by available evidence-based anti-stigma interventions [73].

Early involvement of PWID as opinion leaders and program ambassadors to spread news about SSP may be particularly effective in rural settings with dense social networks, because PWID in these areas tend to trust their friends and family members more than healthcare providers, and news is most efficiently spread via word of mouth. Institutionalizing the role of PWID in SSP service provision, such as formally enlisting them as volunteers or hiring them as paid workers, as well as removing policies prohibiting SSP implementation by local CBOs and grass-root groups representing PWID, may also help convince hesitant PWID to enroll to the program.

Friendly and non-judgmental treatment of PWID, low threshold enrollment practices (e.g., no requirements for picture ID, insurance, residency proof, mandatory HIV testing, etc.) and maintaining clients’ confidentiality will further boost the program’s reputation among PWID networks that can catalyze enrollment, as shown by our study and elsewhere [74–76]. Further, the programs should prioritize coverage of PWID with children and other individuals highly susceptible to stigma and legal repercussions, perhaps by increasing awareness of SSP confidentiality protections and expanding secondary syringe exchange. Multimodal service provision, including satellite and secondary syringe exchange, pharmacy-based and mobile SSPs, and novel syringe sources such as syringe vending machines, may help programs to reach PWID at various stages of readiness to visit the program [10, 57, 58, 77, 78]. These meso-level interventions need to be accompanied by macro-level reforms addressing structural stigma against PWID as captured by us and other studies in rural Appalachia [10, 20, 55]. These reforms should include decriminalization of drug possession and injecting equipment possession, removing any burdensome and unjustified requirements for establishing and operating SSPs and mandating non-prescription sales of syringes at pharmacies. More research is needed on how these meso- and macro-level changes affect the enrollment of PWID into rural SSPs as well as on other SSP implementation and program outcomes, such as continuous attendance, reaching all PWID who need SSP services and meeting their needs in services.

### Conclusions

As rural counties in Kentucky expanded SSP services at an unprecedented rate, little was known about how stigma could affect the pace of PWID enrollment into the programs in rural areas with no prior history of SSP operation. We found, however, that the relationship of stigma to enrollment appeared to vary by features of the social and healthcare environments (e.g., PWUD networks, organization of SSP services) and individual characteristics of PWID (e.g., gender, child custody and social status). SSPs opening in locations with high stigma against PWID need to ensure low threshold and friendly service provision, protect their clients from police and mobilize PWID networks to promote rapid enrollment. Broader legal and policy reforms may be needed to lower barriers for SSP enrollment.

## Supplementary Information

**Additional file 1**. Additional quotes from the participants.

## Data Availability

The interview transcripts are not publicly available for confidentiality reasons.

## Abbreviations

HCV: Hepatitis C virus
HIV: Human immunodeficiency virus
IREF: Intersectional Risk Environment Framework
PWID: People who inject drugs
SSP: Syringe service program

## Footnotes

1 Throughout the text we refer to syringes as a set of a syringe and needle.

2 Here and below we use numbered superscripts to reference additional quotes compiled in the Additional file 1.
