# Supplementary material for: Factors that influence enrollment in syringe services programs in rural areas: a qualitative study among program clients in Appalachian Kentucky
Source: Harm Reduct J. 2021 Jun 30;18:68. doi: 10.1186/s12954-021-00518-z (PMC8244225; doi:10.1186/s12954-021-00518-z)
Supplement: Supplementary file 1 — Additional file 1. Additional quotes from the participants. [file 12954_2021_518_MOESM1_ESM.docx]

**Supplemental materials**

**Additional quotes from the participants**

Q1.

*Participant (P): There are a lot of diabetics that sell their syringes. That's where I would always get them at before. There are a lot of diabetics that use drugs, but I've never seen a diabetic use drugs and have them shoot up, too for some reason. I don't know why. I swear I've never. But I've known a lot of diabetics that trade off their syringes for drugs.*

*Interviewer (I): Do they sell them for money too? How much, about? What's the cost of –*

*P: They'd probably go for $5 apiece.*

- ***a man from county A***

Q2.

*P: You know, at Walmart you can buy a whole box for thirteen dollars and [drug dealers] charge five dollars a thing.*

*I: You mean if you buy it from a dealer?*

*P: Say, when you go buy a pill and you say, “hey I need a needle.” It’s five dollars from them and they are go get them for thirteen dollars at Walmart. But the Walmart is like an hour and a half away. So, I came down here.*

- ***a man from county E***

Q3

*When people find out you're a drug user, and they kind of treat you a certain way, you know. They don't – they look at you as though you're a bad person, or – they either think of you as a bad person, or they think of you as a thief or something like that. But I mean, personally, I don't steal nothing off anybody. And if I want to have a habit, then I support my own habit by either working, or you know, hustling, or whatever I got to do.*

- ***a man from county B***

Q4

*P: [SSP] wasn’t something I wanted to come to. It was just something that – I thought it was a very good thing to do, by all means; but it’s a taboo with it as well. […] I finally started about six months in [after hearing about SSP]. A really good friend of mine told me about [SSP]. I still struggle with coming in here, just because you might see people you know. I don’t like that very well at all. […] I couldn’t make myself come, actually; and then one day I decided to try it, and I came without letting anybody know. I just came hoping for the best, that there wouldn’t be a lot of people. I didn’t know what to expect. I came, and then I told [my boyfriend] about it; and then he started coming, too.*

*I: Then was there anything that made you excited about the possibility of going to the program?*

*P: Just the fact of – there was nothing exciting to me, just for the fact of the stigma, I guess. I struggle with that to this day, but yes; the fact of having clean syringes and making sure things are as safe as they can be –*

*I: Okay. So, you mentioned stigma. That was your primary concern. Could you explain why a little bit more?*

*P: Just the way – this is a very small town. I’ve lived here my entire life. Seeing people I know – they try to keep it as confidential as they can; but at this point I’m sure everyone knows what’s going on here on Tuesday, or whatever day it is. Just the fact of running into – I just find it really embarrassing. I find it mortifying, really. So, I try not to come as much as possible, but I quit coming for myself for quite some time. I was doing it for my brothers, because they’re terrible. I just couldn’t get them to come do this.*

- ***a woman from county A***

Q5

*P: [SSP] wasn’t something I wanted to come to. It was just something that – I thought it was a very good thing to do, by all means; but it’s a taboo with it as well. […] I finally started about six months in [after hearing about SSP]. A really good friend of mine told me about [SSP]. I still struggle with coming in here, just because you might see people you know. I don’t like that very well at all. […] I couldn’t make myself come, actually; and then one day I decided to try it, and I came without letting anybody know. I just came hoping for the best, that there wouldn’t be a lot of people. I didn’t know what to expect. I came, and then I told [my boyfriend] about it; and then he started coming, too.*

*I: Then was there anything that made you excited about the possibility of going to the program?*

*P: Just the fact of – there was nothing exciting to me, just for the fact of the stigma, I guess. I struggle with that to this day, but yes; the fact of having clean syringes and making sure things are as safe as they can be –*

*I: Okay. So, you mentioned stigma. That was your primary concern. Could you explain why a little bit more?*

*P: Just the way – this is a very small town. I’ve lived here my entire life. Seeing people I know – they try to keep it as confidential as they can; but at this point I’m sure everyone knows what’s going on here on Tuesday, or whatever day it is. Just the fact of running into – I just find it really embarrassing. I find it mortifying, really. So, I try not to come as much as possible, but I quit coming for myself for quite some time. I was doing it for my brothers, because they’re terrible. I just couldn’t get them to come do this.*

- ***a woman from county A***

Q6

*I: Do you remember when you heard about the program and who told you about it?*

*P: Yeah, and I told him that that’s horse shit. That there wasn’t no such program. And I said if they are, they’re keeping tabs on [SSP clients]. You know, I’m naturally paranoid anyway, but as I said if there is, I’m not going. […] And [my friends are] like, why? I said, because they document that stuff. And who’s to say who’s ending up with that information?*

*I: […] So, that was your first time hearing about [SSP]?*

*P: Yeah, and then it took me a long time to come up here, because I felt like I was being judged.*

***- a woman from county E***

Q7

*I: Did you have any concerns about coming to the program before you came?*

*P: Not really, because it is confidential; so not really.*

*I: Yes. Did you know that before you came to the program?*

*P: Yes.*

*I: How did you hear about that?*

*P: The woman up front [at the county health center] – whenever I talked to her, whenever [SSP] was going on – she had mentioned that it was confidential. At first, I didn’t want to come; but when she said that, then I wanted to come.*

*I: Had you come to the health department for something else, or what –*

*P: Yes, I usually come here for a checkup.*

*I: So, at that point she told you about the program.*

*P: Yes.*

- ***a woman from county C***

***Q8***

*I: Before going [to the program for the first time], is there anyone you voiced your concerns to, or talked to about going?*

*P: Yeah, my friend Jamie who passed away. […] He assured me that … He’s always pretty cool when it all confidential. But he really didn’t have anything to lose either. At that time, I did have children, so I couldn't let anybody know what I did. I kept it very, very private.*

- ***a man from county A***

Q9

*I: Oh okay, so then do you think some people might be worried about losing access to like other services?*

*P: Mm-hmm, because at first like I said when I had my kid it was like no, no way they're going to see me with my child. You know, change ain't going to happen.*

*I: So then what do you think could be done to make it easier for them to come?*

*P: I don't know that's a tricky one. If I still had my child I wouldn't be coming, just because I don't know. It's just a thing.*

*I: Do you think there is anything the program could do to--*

*P: They wouldn't convince me ever. Because no, you know, if a case were to come open on me, then no, I would have never just for protection of, I don't know, just no. That's just me, you know, everybody is different. I know a lot of women that have kids that won't go. Cause they come up here to get their, you know, they come up here for their, so.*

- ***a woman from county B***

Q10

*Well…I had one of my friends tell me about it I guess, say about 8 months ago. It might have been a lot longer than that. He came down here and he said “wanna go?” and I’m like nah.. […] But I thought again and I wish I had come down here. Probably seven or eight months [ago], one of my friends told me about it. I mean, he, he… I’ve seen one of them signs up but what I thought, when he told me about it, you know, he talked real good about it. And I still didn’t come down here, then I finally did. And I love it down here really.*

***a man from county E***

Q11

*I: What led you to your decision to finally come out and give [SSP] a try?*

*P: Well actually it was more my buddy’s – he was coming to it, and I kept getting stuff – syringes – from him. I guess he got tired of having to give them away, and he brought me down here with him one day. That way I could get into it and get started and get my own. Plus, he wanted some of them back; because I got them from him. So I done it, and that’s what got me in here my first time – a friend.*

***a man from county A***

Q12

*I: Then how did you get here that first time?*

*P: My husband pushed me.*

*I: Is he the only person that came with you that first time, it was just you and him?*

*P: Yeah.*

- ***a woman from county A***

Q13

*I: How does privacy influence your decision to come to the program?*

*P: A lot because usually, I mean like anybody can come in here if they didn’t have that privacy, say, any and the law and stuff come this, this, this, and then they would have to tell them because any other time, they would have to, but now where they got that signed paper and stuff, they don’t have to tell you. They don’t even get your name. They get your first two letters of your first name and your first two letters of your last name. That’s it. That’s how you make your card. It ain’t got your whole name on the card. It ain’t got your address, birth certificate. I mean it ain’t got all that on it; so, you don’t have to worry about everybody knowing into that. I like that, too, because I mean, God, it took for me and like my name on – no, I wouldn’t have done it.*

- ***a woman from county C***

Q14

*I: Were you unsure about the program at all?*

*P: Not really. I've got other family members that use them in the city. So, I pretty much knew what I was looking at.*

- ***a woman from county C***
